# The amino acid substitutions A30W, K28A, and M35C alter amyloid-β peptide toxicity in cell culture and in an *in vivo* model of amyloidosis in *Caenorhabditis elegans*

**DOI:** 10.3389/fnagi.2026.1843210

**Published:** 2026-06-29

**Authors:** Diana Patricia Quijano-Guerrero, Ana Esther Estrada-Rodríguez, Mauricio Caballero-Rodríguez, Danna Loretta de León-Rivera, Cristina Rodríguez-Padilla, Victor Julian Valdes, Diana Caballero-Hernández, Román Vidaltamayo, Ana Carolina Martínez-Torres, Viviana Zomosa-Signoret

**Affiliations:** 1 Laboratorio de Inmunología y Virología, Departamento de Microbiología e Inmunología, Facultad de Ciencias Biológicas, Universidad Autónoma de Nuevo León, San Nicolás de los Garza, Nuevo León, Mexico; 2 School of Medicine, University of Monterrey (UDEM), San Pedro Garza García, Nuevo León, Mexico; 3 Department of Cell Biology and Development, Institute of Cellular Physiology (IFC), National Autonomous University of Mexico (UNAM), Mexico City, Mexico

**Keywords:** Alzheimer’s disease, amyloid pathology, amyloid-β peptide, amyloid-β variants, Caenorhabditis elegans

## Abstract

The buildup of toxic aggregates formed by the amyloid-β peptide 1–42 (Aβ_42_) is a central process in Alzheimer’s disease (AD) pathology. The peptide’s self-assembly and toxicity are highly dependent on its primary amino acid sequence and can be altered by modifying key residues. Specifically, the single amino acid substitutions A30W, K28A, and M35C can reduce the aggregation and toxicity of the Aβ_42_ peptide. In this study, we further evaluated the effects of these mutations in a C6 rat glioma cell line and in the *Caenorhabditis elegans* strains CL2006 and CL4176, which express muscular Aβ_42_ as an *in vivo* model. Our results showed that the A30W, K28A, and M35C substitutions reduce apoptosis induction in cell culture, in contrast to the WT Aβ42 peptide. In *C. elegans*, the three variants extended the lifespan of CL2006 worms by reducing fibrillar aggregates or altering aging, whereas the M35C peptide delayed the paralysis of CL4176 worms. Additionally, the substitutions altered oxidative stress and autophagy in control worms. Taken together, these results suggest that the A30W, K28A, and M35C substitutions reduce Aβ42 toxicity in cell culture and in *C. elegans* and could protect the nematode against Aβ42 toxicity.

## 1 Introduction

Alzheimer’s disease (AD) is a progressive and irreversible neurodegenerative disorder that represents the most common cause of dementia. Although its etiology remains controversial, evidence suggests that the accumulation of misfolded amyloid-β (Aβ) peptides triggers AD pathology (Barage and Sonawane, 2015; Hardy and Higgins, 1992). Aβ peptides are derived from the processing of the amyloid precursor protein (APP) via the so-called amyloidogenic pathway, where APP is subsequently cleaved by the β and γ-secretases (O’Brien and Wong, 2011). After synthesis, Aβ can enter a process of self-aggregation in which misfolded monomers form oligomeric and fibrillar aggregates (Finder and Glockshuber, 2007). The conformation of Aβ monomers appears to be partially unfolded with at least one α-helix around residues 13–23, and a β-turn structure within residues 21–30 flanked by two regions with a tendency to form β-sheets (Lin et al., 2019; Vivekanandan et al., 2011). An increase in the β-sheet content precedes the assembly of multiple Aβ monomers into a nucleus that allows the formation of oligomers and fibrils through monomer addition (Niu et al., 2024; Yang and Teplow, 2008). A secondary nucleation, in which pre-existing fibrils act as a scaffold for the formation of new aggregates, further accelerates and spreads the process (Niu et al., 2024). These Aβ aggregates are toxic to neurons. Amyloid plaques, formed by the deposition of fibrillar Aβ, induce abnormalities and atrophy in neighboring neurons (Tsai et al., 2004). Moreover, Aβ oligomers interact with multiple receptors, leading to cell death, and can directly induce oxidative stress, cell membrane disruption, mitochondrial dysfunction, and autophagy impairment (Guglielmotto et al., 2014; Tolar et al., 2021; Vander Zanden et al., 2019).

The aggregation of Aβ is highly dependent on its primary amino acid sequence. Cleavage of APP yields different Aβ isoforms, with Aβ_42_ presenting an additional isoleucine and an alanine at the C-terminus, in contrast to the most abundant isoform Aβ_40_. The extra residues in Aβ_42_ increase its tendency to form β-sheet structures, facilitating its aggregation and enhancing neurotoxicity (Lin et al., 2019; Qiu et al., 2015). Furthermore, several mutations within the Aβ sequence can predispose against AD by accelerating or stabilizing the toxic aggregates, such as the Arctic missense mutation E22G (Julia and Goate, 2017). However, other changes to the Aβ sequence can have beneficial effects. The positively charged lysine at position 28 stabilizes the β-turn by forming a salt bridge with the Asp23 residue (Petkova et al., 2002; Shuaib et al., 2020). The substitution of Lys28 with a hydrophobic alanine (K28A) reduces the peptide’s β-sheet content, altering its aggregation (Shuaib et al., 2020; žganec et al., 2016). Similarly, the alanine at residue 30 participates in the formation of the β-turn, which can be destabilized when substituting for a tryptophan (A30W) (Estrada-Rodríguez et al., 2019). The methionine at position 35 is also relevant for aggregation and is mainly associated with Aβ-induced oxidative stress, which can be prevented by its substitution with cysteine (M35C) (Yatin et al., 1999). Previously, these three variants showed reduced fibrillar aggregate formation and interacted with Aβ_42_ *wildtype* to inhibit its aggregation *in vitro* (Estrada-Rodríguez et al., 2019). To further characterize this potential therapeutic effect, we aimed to study the impact of the A30W, K28A, and M35C substitutions on Aβ_42_ toxicity in cell culture in an *in vitro* model of glioma and in an *in vivo* AD model.

The free-living nematode *Caenorhabditis elegans* is a widely used model organism for neurodegenerative diseases. The transgenic strains CL4176 and CL2006 have been developed to express the human Aβ_42_ peptide in muscle cells and exhibit distinct pathological phenotypes, including progressive paralysis. Nematodes are also highly used to perform high-throughput drug screening to identify chemical compounds with therapeutic activity (Link, 1995; Lublin and Link, 2013). In this work, we treated *C. elegans* with A30W, K28A, and M35C peptides to assess their effects, with or without endogenous Aβ_42_ expression.

## 2 Materials and methods

### 2.1 Synthetic peptides

Peptides, were acquired from GenScript (Piscataway, NJ, United States) including Aβ_42_ WT (purity > 95%) and the Aβ_42_ variants A30W, K28A, and M35C (purity < 85%). All peptides were diluted to 40 μM in PBS and incubated for 96 h at 37°C and 340 rpm before assays.

### 2.2 Cell culture

C6 rat glioma cell line (CCL-107, ATCC, Manassas, VA., United States) was cultured in Dulbecco’s Modified Eagle Medium (Caisson, North Logan, UT, United States) supplemented with 2.5% fetal bovine serum (GIBCO, Grand Island, NY, United States). Cells were plated at 50% confluence in a 96-well plate and grown for at least 48 h before assays. Cultures were maintained at 37°C and 5% CO_2_.

### 2.3 Cell death assays

Cell viability, cytotoxicity, and apoptosis evaluations were performed using the ApoTox-Glo Triplex Assay (Promega). Cell cultures were treated for 24 h with or without the preincubated peptide samples. The reagent containing the GF-AFC and the bis-AAF-R110 substrates was added to each well, and fluorescence was measured after 30 min using a GloMax microplate reader. Excitation and emission were set to 400 and 505 nm for viability measurements, and to 485 and 520 nm for cytotoxicity measurements. For apoptosis, the Caspase-Glo^®^ 3/7 reagent was added, and luminescence was measured after 30 min.

### 2.4 Flow cytometry

Annexin−V−allophycocyanin at 0.1 μg/mL (BD Pharmingen, San Jose, CA., United States) and propidium iodide at 0.5 μg/mL (Sigma−Aldrich) were used to measure phosphatidylserine exposure and cell viability of a total population of 10,000 cells in a BD Accuri C6 flow cytometer (Biosciences, Franklin Lakes, NJ, United States).

### 2.5 Autophagy flux

Cell cultures were treated for 24 h with or without the preincubated peptide samples. Autophagy activity was evaluated using the Autophagy Detection Kit (ab139484, Abcam). Cells were incubated with the green detection reagent and a nuclear stain, and fluorescence was measured using a GloMax microplate reader with excitation at 475 nm and emission at 500–550 nm. As a positive control, cells were treated with 0.5 μM rapamycin (#R-5000, LC Laboratories, Woburn, MA) and 40 μM chloroquine for 24, 4 h before the experiments were completed.

### 2.6 Plasmids

Expression cassettes were designed for Aβ_42_ and the Aβ_42_ variants A30W, K28A, and M35C, codon-optimized for expression in *Escherichia coli* (Walsh et al., 2009). Each sequence was synthesized and cloned into a pET3a vector between the NdeI and BamHI sites by Gene Universal (Newark, DE, United States). All vectors were transformed into *E. coli* BL21(DE3)pLysS Ca2+ -competent cells by heat shock and spread on LB agar plates supplemented with 100 μg/mL of ampicillin and 30 μg/mL of chloramphenicol (Sigma-Aldrich).

### 2.7 *C. elegans* strains maintenance

The *C. elegans* transgenic strains with expression of Aβ42 in muscle CL2006 (dvIs2 [pCL12(unc-54/human A-Beta(1–42) minigene) + rol-6(su1006)]), and CL4176 (dvIs27 [myo-3p::A-Beta (1-42)::let-851 3’UTR) + rol-6(su1006)]), the control strain CL802 (smg-1(cc546) I; rol-6(su1006) II) and the transgenic strain AMH43 were acquired from the Caenorhabditis Genetics Center (University of Minnesota, United States). Nematodes were grown at 16 °C on plates containing solid nematode growth medium (NGM) seeded with *E. coli* strain OP50 for maintenance. Age-synchronized populations were obtained by allowing gravid hermaphrodites to lay eggs for 4–6 h.

### 2.8 Peptide expression and *C. elegans* treatment

Using a single colony of transformed *E. coli*, 3 mL of LB medium containing 100 μg/mL of ampicillin and 30 μg/mL of chloramphenicol was inoculated and grown overnight at 37°C and 200 rpm. Bacterial cultures were diluted with fresh LB medium to an OD600 nm of 0.5, and peptide expression was induced by adding 1 mM isopropyl β-D-thiogalactopyranoside (UltrapureTM IPTG, Invitrogen) and incubating at 37°C. Four hours after induction, 100 μL of the bacterial culture was spread onto NGM plates for nematode treatment.

### 2.9 Lifespan assays

Synchronized CL2006 and CL802 eggs were grown at 16°C on NGM plates seeded with *E. coli* expressing each peptide or *E. coli* transformed with an empty pET3a plasmid as a control. Nematodes were scored daily using a platinum wire to check for a response after gentle prodding. Worms were transferred daily to a fresh plate until they ceased to lay eggs, and thereafter only once a week, with fresh bacterial culture supplemented every 2 days (Amrit et al., 2014).

### 2.10 Aβ aggregates staining

Synchronized CL2006 and CL802 eggs were grown at 20°C on NGM plates seeded with each treatment. Five-day-old worms were collected, washed with M9 buffer, and fixed in 70% ethanol for 2 h. The fixed nematodes were stained with 0.125% thioflavin S (Sigma-Aldrich) in 50% ethanol. After an hour, the worms were destained by washing with ethanol and mounted. Fluorescence images were obtained using a Zeiss fluorescence microscope. The number of thioflavin S-positive deposits in the worm’s head was counted.

### 2.11 Paralysis assays

Synchronized CL4176 and CL802 eggs were grown at 16°C on NGM plates seeded with each treatment. After 36 h of incubation, the temperature was upshifted to 25°C to induce transgene expression. Paralysis was scored every 2 h starting at 36 h of induction. Nematodes that did not move or moved only the head after being gently touched with a platinum wire were scored as paralyzed.

### 2.12 Reactive oxygen species (ROS) measurement

Synchronized CL4176 and CL802 eggs were grown in NGM plates seeded with each treatment at 16°C for 36 h and then upshifted to 25°C for 36 h. After incubation, nematodes were collected, washed with PBS, and 50 nematodes per replica were transferred into a 96-well plate containing 200 μL of PBS, 0.01% Tween-20, and 50 mM of 2’, 7-dichlorofluorescein diacetate (H2DCF-DA, Invitrogen). Fluorescence was quantified at 485 nm excitation and 530 nm emission every 30 min for 3 h using a GloMax microplate reader (Zhang et al., 2016).

### 2.13 *In vivo* autophagy

Synchronized AMH43 eggs were grown in NGM plates seeded with each treatment at 16°C. Five- and ten-day-old worms were collected, washed with M9 buffer, anesthetized with 100 mM sodium azide, and mounted on 5% agarose pads. Fluorescence images were obtained using a Zeiss fluorescence microscope, and quantification of GPF: LGG-1 positive puncta was carried out using the “analyze particle” plug-in from ImageJ software with a size from 1 to 20 μm^2^ and circularity of 0.1–1.

### 2.14 Daf-16 localization

Synchronized TJ356 eggs were grown in NGM plates with each treatment at 16°C. Five-day-old worms were collected, washed with M9 buffer, anesthetized with 100 mM sodium azide, and mounted on 5% agarose pads. Fluorescence images were obtained using a Zeiss fluorescence microscope. Worms were categorized as cytosolic, intermediate, and nuclear. Nuclear localization was scored when nuclear fluorescence dots appeared throughout the entire body from head to tail. Intermediate localization was scored when there was obvious visible nuclear localization, but not as complete. When no nuclear fluorescence dots were observed, cytosolic localization was scored. The number of worms in each category was counted (Oh et al., 2005; Wang et al., 2015; Zhao et al., 2017).

### 2.15 Statistical analysis

Statistical analysis was performed using GraphPad Prism 11 (San Diego, CA, United States). Significance was assessed by a one-way ANOVA test followed by Dunnett’s multiple comparisons test to compare with the control group, or by Tukey’s multiple comparisons test to compare with all groups, including the control. Lifespan and paralysis data were analyzed using the Kaplan-Meier estimator, and curves were compared using the log-rank (Mantel–Cox) test (Amrit et al., 2014).

## 3 Results

### 3.1 The Aβ_42_ variants reduced cytotoxicity and apoptosis in cell culture

**FIGURE 1:**
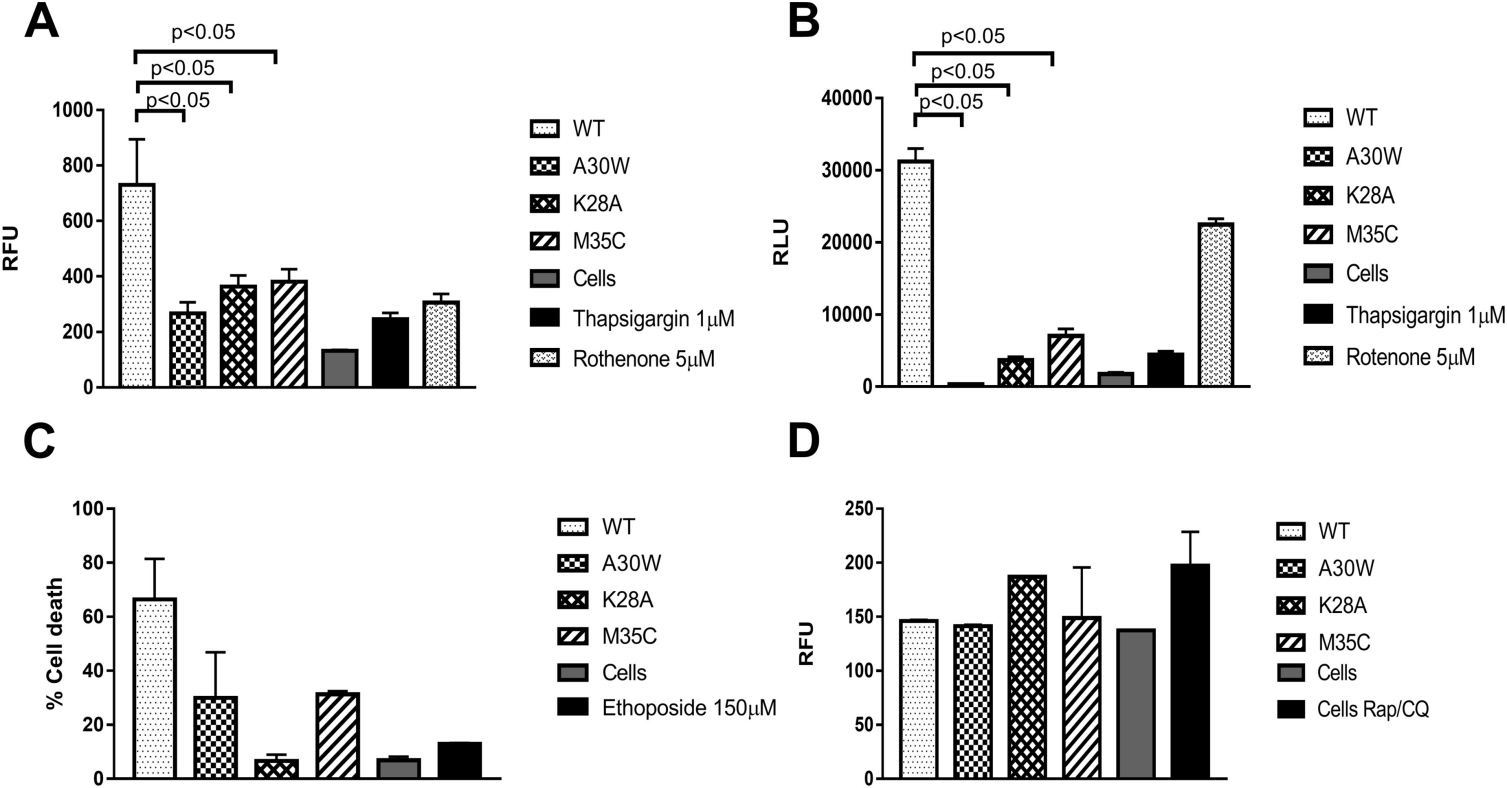
Toxicity of Aβ42 peptides on cell culture. **(A)** Cytotoxicity assay on C6 glioma cells with or without treatment with Aβ42 peptides. Cells were plated in 96-well plates at 20 × 10^3^ per well. Two wells were tested for each treatment. **(B)** Luminescence assay to evaluate apoptosis in response to treatment. Cells were plated in 96-well plates at 20 × 10^3^ per well. Two wells were tested for each treatment. **(C)** Flow cytometry AV/PI assay performed to confirm apoptosis in response to treatments. Cells were plated in 24-well plates at 20 × 10^4^ per well. Two wells were tested for each treatment. **(D)** Autophagy flux evaluation in response to treatments. Cells were plated in 96-well plates at 20 × 10^3^ per well. Two wells were tested for each treatment. Error bars indicate SEM. Statistical differences obtainedby Dunnett’s multiple comparison.

We evaluated cytotoxicity and apoptosis in C6 glioma cells after treatment with the Aβ_42_ peptides. The A30W, K28A, and M35C variants decreased cytotoxicity compared to the WT peptide (Figure 1A). Consistently, the WT peptide, but not the variants, increased cell apoptosis (Figure 1B).

To further confirm the effect on apoptosis, we performed flow cytometry using Annexin V-allophycocyanin/propidium iodide (AV/PI). During apoptosis, phosphatidylserine, a phospholipid usually restricted to the inner cell membrane, is translocated to the outer membrane as a signal for the clearance of the apoptotic cell (Shlomovitz et al., 2019). The fluorescently labeled Annexin V binds to exposed phosphatidylserine, whereas propidium iodide cannot enter apoptotic cells, allowing differentiation from necrotic cells (Crowley et al., 2016). AV/PI results also showed decreased apoptosis after treatment with the three variants compared with the WT peptide (Figure 1C).

Autophagy flux analysis showed that only the K28A variant led to a slight increase in autophagy levels, which was not statistically significant (Figure 1D).

### 3.2 The Aβ_42_ variants protected CL2006 nematodes

To facilitate the treatment of *C. elegans*, we expressed each peptide in *E. coli* and fed it to the nematodes. Similar amyloid proteins are internalized through the intestine and induce systemic effects (Chen et al., 2016; Perni et al., 2021). Specifically, feeding Aβ-expressing *E. coli* results in intestinal membrane damage, facilitating its internalization via endocytosis (Hassan et al., 2015; Julien et al., 2018; Shea et al., 2019).

**FIGURE 2:**
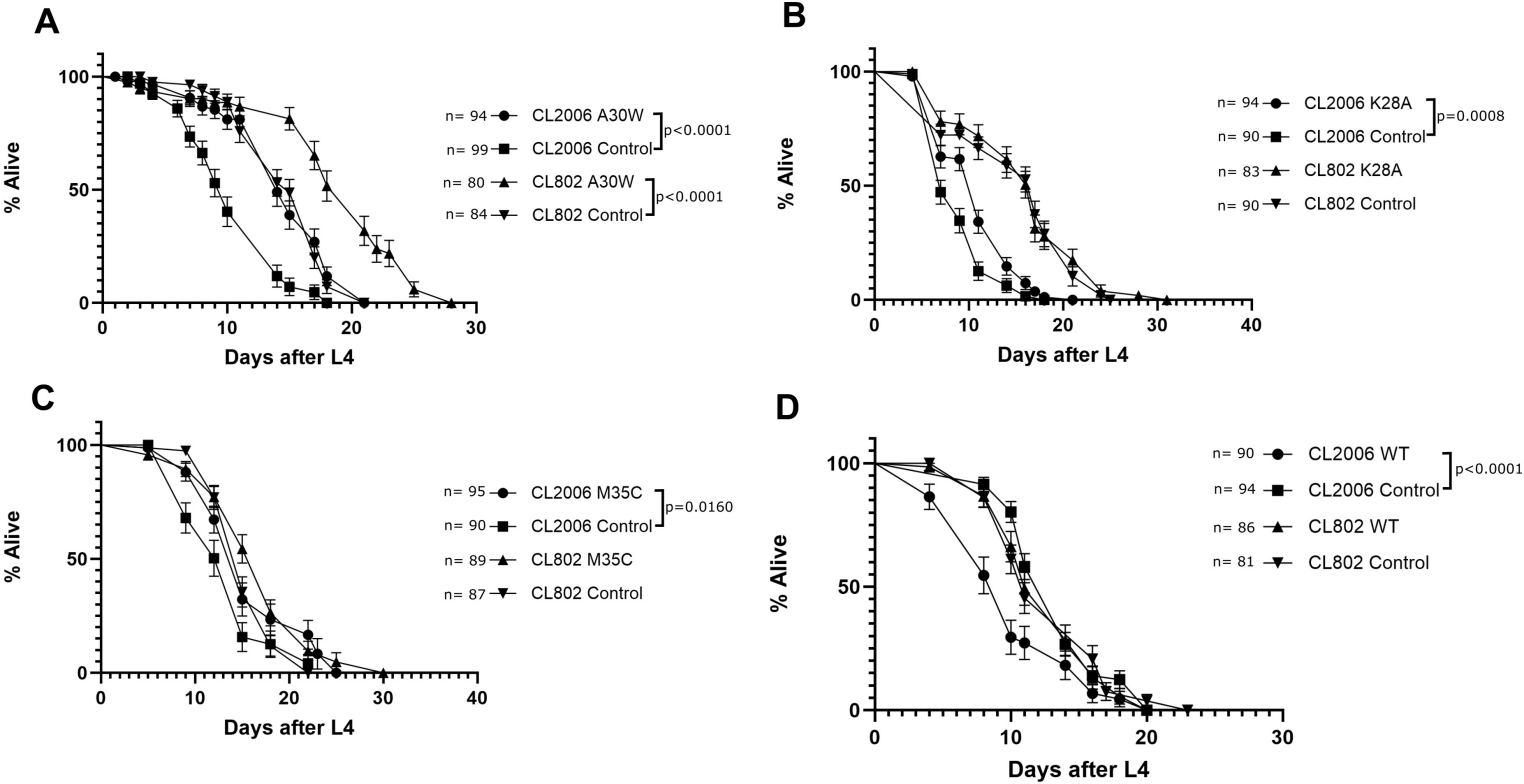
Effects on the lifespan of CL2006 nematodes. **(A–D)** Lifespan analysis of CL2006 worms and the control strain CL802 treated with Aβ42 variants or control. The data correspond to three independent experiments, (∼ 30 nematodes per replica). Error bars represent SE. Statistical differences obtained by log rank tests.

We evaluated the effect of Aβ_42_ peptides on the viability of CL802 and CL2006 nematodes and the paralysis onset in CL4176 nematodes. Both CL2006 and CL4176 transgenic strains express Aβ_42_ within muscle cells, resulting in systemic defects like shorter lifespan and paralysis (Lublin and Link, 2013). Treatment with A30W peptide significantly extended the lifespan of CL2006, showing a survival curve similar to the control strain CL802 without treatment (Figure 2A). Unexpectedly, A30W was also able to increase the lifespan of CL802 worms that do not produce Aβ, indicating a protective but nonspecific effect. The K28A and M35C variants extended the lifespan of CL2006 nematodes but did not affect CL802 worms (Figures 2B,C). The WT, in contrast to the variants, decreased the lifespan of CL2006 worms (Figure 2D).

**FIGURE 3:**
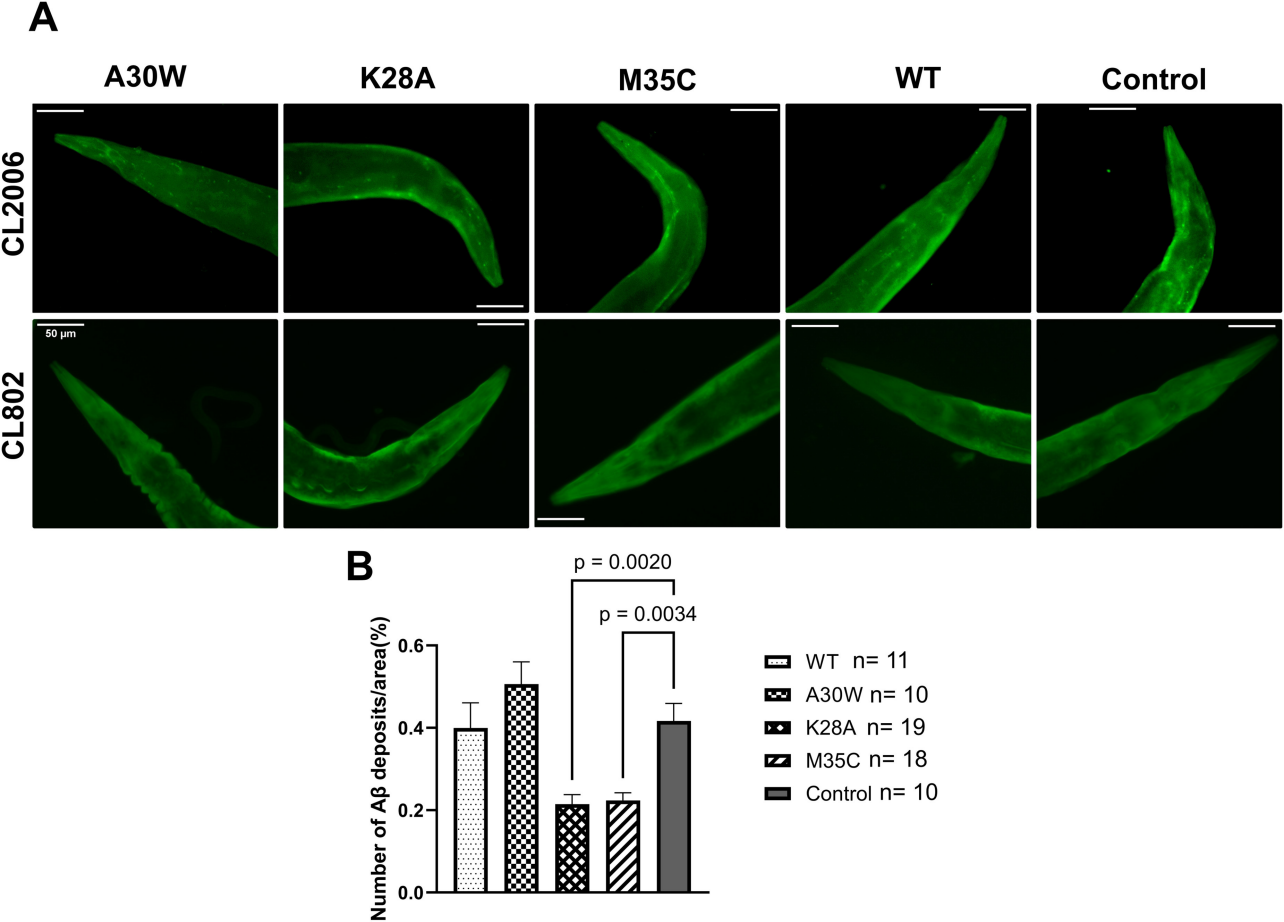
Aβ deposits on CL2006 nematodes in response to the treatments. **(A)** Representative images of CL2006 and CL802 worms stained with thioflavin S after treatment with Aβ42 variants or control. **(B)** Quantification of Aβ deposits in the head. Data corresponds to three independent experiments (∼ 3 nematodes per replica). Error bars indicate SEM. Statistical differences obtained by Tukey’s multiple comparison.

To assess the effect of our treatments on amyloid fibril formation, we scored the number of Aβ deposits in the head area of CL2006 worms stained with thioflavin S (Figure 3A). Treatment with the K28A and M35C variants reduced the number of Aβ aggregates. In contrast, the A30W and WT peptides did not differ from the control group. In the control strain CL802, none of the treatments resulted in the appearance of amyloid deposits (Figure 3B).

### 3.3 The M35C variant delays paralysis, and K28A reduces ROS in CL4176 nematodes

**FIGURE 4:**
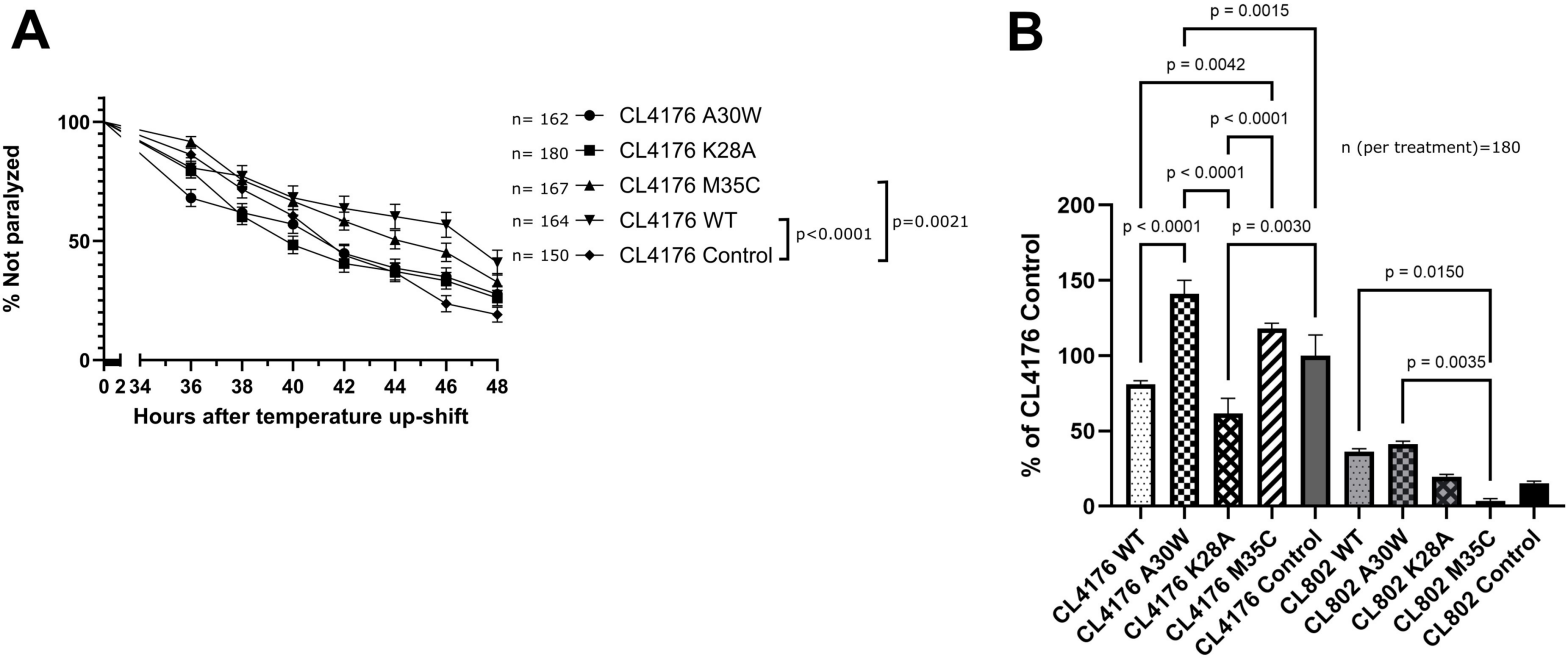
Effects on paralysis and the oxidative stress of CL4176 nematodes. **(A)** Paralysis assay in CL4176 worms with and without treatment. The data corresponds to six independent experiments (∼ 30 nematodes per replica). Error bars represent SE. Log-rank tests showed significant differences between WT and control (*p* < 0.0001), and M35C and control (*p* = 0.0021). **(B)** Fluorescence signal of H2DCFDA in CL4176 and CL802 treated with Aβ42 variants or control. Results represent four independent experiments (∼50 nematodes per replica) and are expressed as a percentage of fluorescence relative to the CL4176 control, which is set at 100%. Error bars indicate SEM. Statistical differences obtained by Tukey’s multiple comparison.

CL4176 shows a more acute phenotype than CL2006, with paralysis onset only 32 h after temperature upshift (Drake et al., 2003). Treatment with A30W and K28A failed to significantly alter the paralysis of CL4176 worms compared to the control (Figure 4A). In contrast, the M35C variant and, unexpectedly, the WT peptide significantly delayed the onset of paralysis.

Additionally, CL4176 nematodes exhibit a significant increase in oxidative stress following the induction of Aβ expression (Drake et al., 2003). Therefore, we evaluated ROS induction in CL802 and CL4176 nematodes following treatment with Aβ_42_ peptides (Figure 4B). Treatment with the A30W peptide significantly increased oxidative stress in Aβ worms compared to WT, K28A, and control. K28A reduced oxidative stress compared to control, whereas M35C and WT did not show significant changes. Treatment with WT, unexpectedly, displayed less oxidative stress when compared with A30W and M35C peptides. In CL802 worms, treatment with M35C reduced oxidative stress, but was only significant when compared to WT and A30W.

### 3.4 The M35C and A30W variants altered autophagy *in vivo*

**FIGURE 5:**
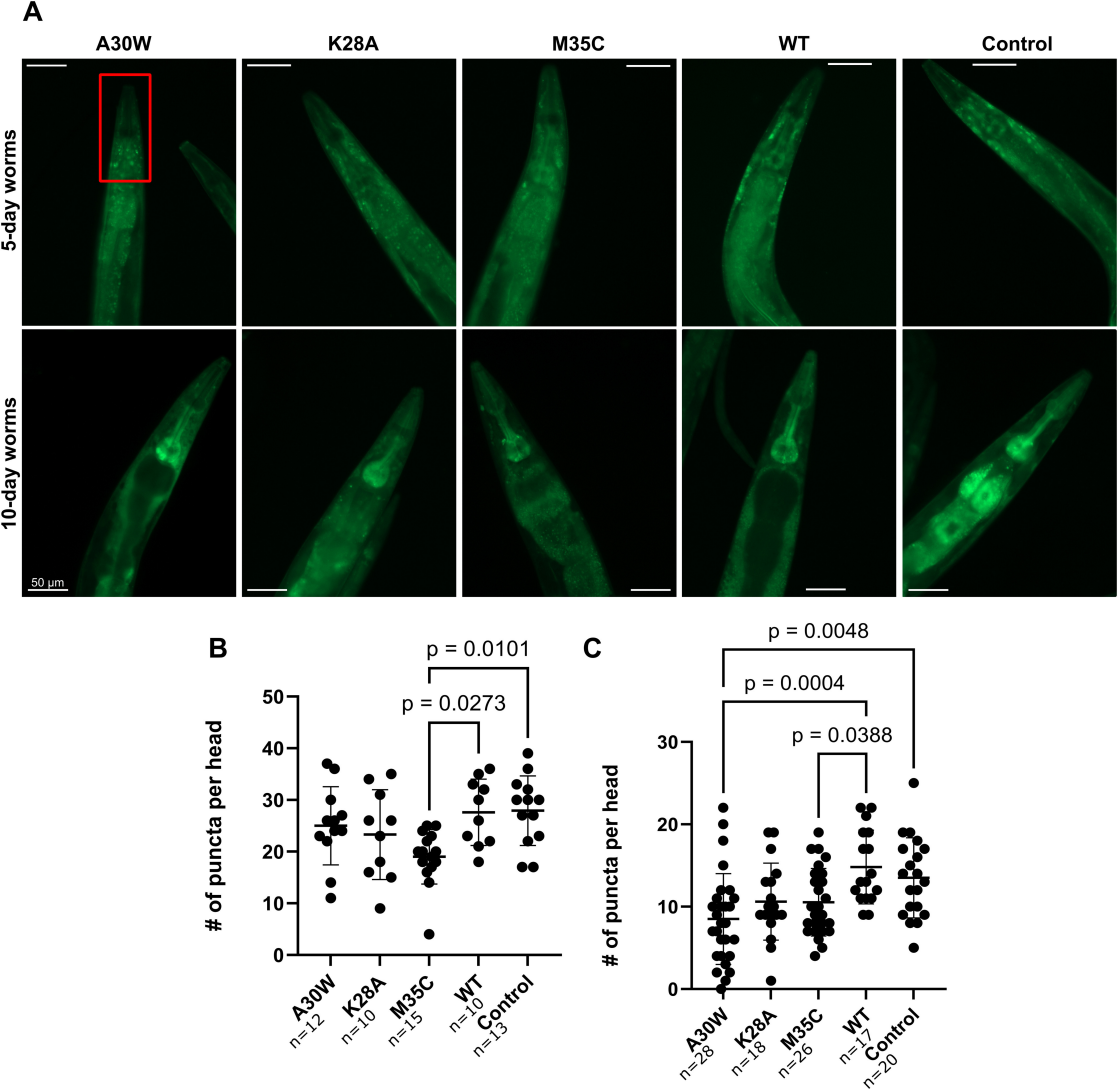
Changes in autophagy in *C. elegans*. **(A)** Representative images of five and ten-day-old AMH43 nematodes treated with Aβ42 variants or control. **(B,C)** Quantification of GPF::LGG-1 positive puncta in the head, delimited by the end of the pharynx (red area). Data comes from three independent experiments (∼3 nematodes per replica). Error bars indicate SEM. Statistical differences obtained by Tukey’s multiple comparison.

To study autophagy *in vivo*, we treated AMH43 nematodes expressing the GPF: LGG-1 reporter. LGG1 is a ubiquitin-like protein belonging to the ATG8 family. Like its orthologs, LGG-1 is recruited to the inner and outer membranes of the forming autophagosome, allowing its use as a marker of autophagy activity in *C. elegans* (Leboutet et al., 2023; Zhang et al., 2015). For 5-day nematodes, we observed similar formation of GPF::LGG-1-positive puncta in the hypodermis, pharynx, and neurons across all group treatments. In contrast, 10-day worms showed signal mostly restricted to the pharynx (Figure 5A). Quantification of the puncta around the head area revealed a tendency to a smaller number in nematodes treated with the Aβ_42_ variants compared to the control and WT peptide; however, only M35C showed a significant difference at both times, and A30W at 10-days (Figures 5B,C).

### 3.5 The peptides can alter the localization of DAF-16 in *C. elegans*

**FIGURE 6:**
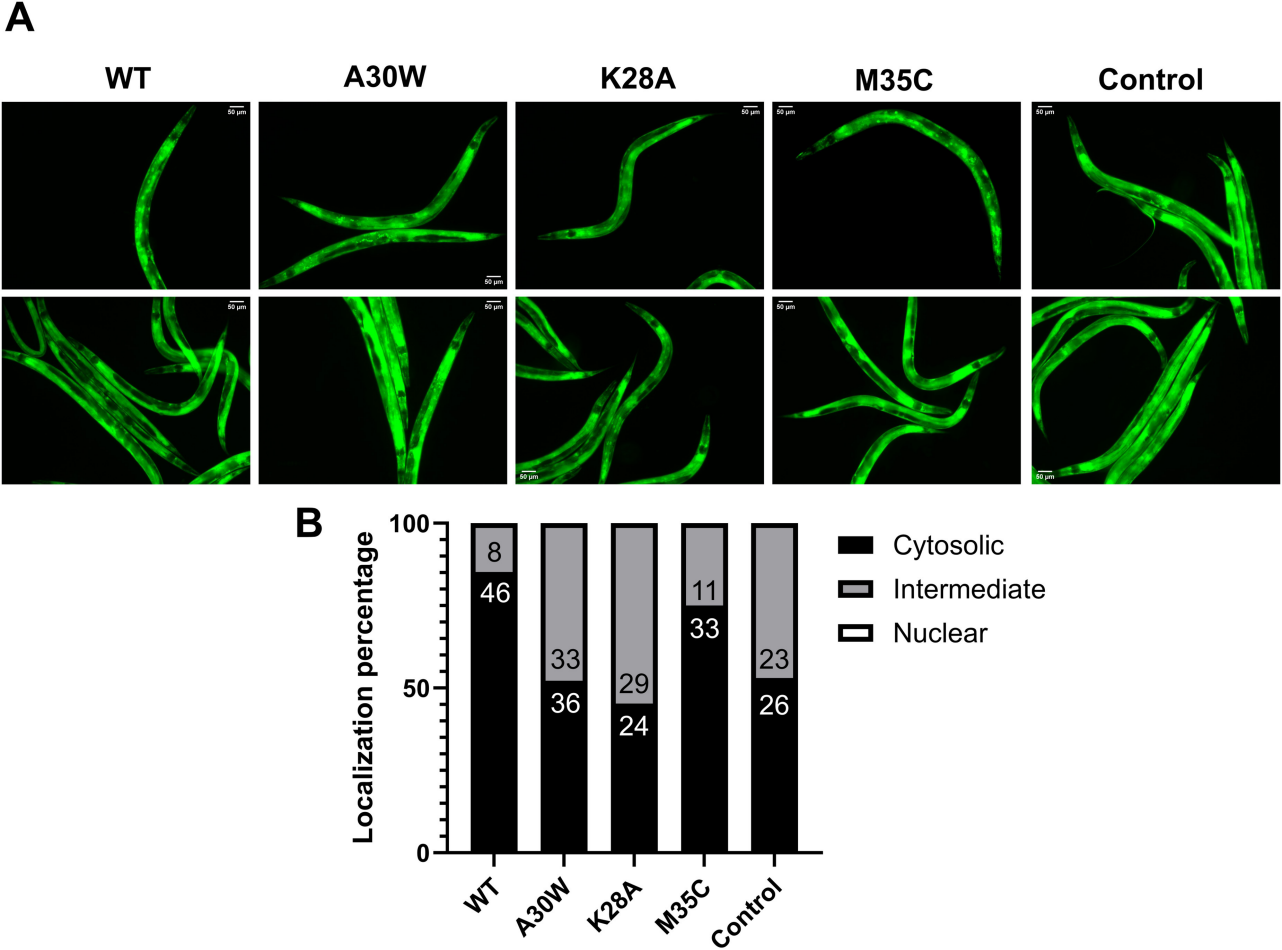
Subcellular localization of DAF-16 in the nematode. **(A)** Representative images of five and 10-day-old TJ356 nematodes treated with Aβ42 variants or control. **(B)** Distribution of the DAF-16: GFP localization phenotype. Numbers in bars represent the quantity of nematodes classified in each category. Data comes from three independent experiments (∼16 nematodes per replica). No difference between groups was observed by Tukey’s multiple comparison.

In the adult worm, the FOXO transcription factor DAF-16 is activated during aging by translocating into the nucleus, thereby increasing longevity and stress resistance (Lin et al., 2001; Tissenbaum, 2018). The constitutive activation of DAF-16 can extend the lifespan of wild-type nematodes up to three times as long as usual (Lin et al., 2001). To evaluate whether the lifespan extension induced by our treatments could be related to the activation of DAF-16, we treated TJ356 nematodes that express DAF-16: GFP. We observed that the WT and M35C increased the retention of the transcription factor in the cytosol (Figure 6B). Although the A30W and K28A variants showed no significant difference in the distribution of DAF-16 to when compared to the control, the nuclear accumulation was more evident in the experimental groups (Figure 6A).

## 4 Discussion

The accumulation of Aβ_42_ is believed to be the primary driver of Alzheimer’s pathology. The emergence of toxic forms of Aβ_42_ is highly dependent on its primary sequence through the process of aggregation, as modification of specific amino acidic residues can alter Aβ_42_ toxicity (Yu et al., 2024). We observed that the A30W, K28A, and M35C substitutions reduced the cytotoxicity and apoptosis induced by the peptide in C6 glioma cells. The K28A and M35C variants showed an extension in the lifespan of CL2006 nematodes, accompanied by a reduction of toxic fibrils. The A30W peptide also increased CL2006 worms’ lifespan, but without altering Aβ aggregation. Additionally, M35C and the WT peptide delayed paralysis, and K28A reduced oxidative stress in CL4176 nematodes. Moreover, in nematodes without Aβ_42_ expression, the A30W variant also extended lifespan, M35C reduced oxidative stress, and both peptides altered autophagy.

The results obtained in this study confirm our previous observations on the reduction of cytotoxicity due to the amino acid substitutions (Estrada-Rodríguez et al., 2019) and revealed that it is mainly associated with reduced apoptosis. Multiple studies have reported that treatment with Aβ_42_ induces apoptosis in cell culture through the activation of caspase-8, caspase-9, and caspase-3 (Awasthi et al., 2005; Islam et al., 2017; Takada et al., 2020). The apoptosis induction appears to be dependent on the formation of fibrillar aggregates, as it is reduced in the absence of polymerization (Islam et al., 2017; Takada et al., 2020; Wogulis et al., 2005). The A30W, K28A, and M35C peptides display less tendency to aggregate into fibrils (Estrada-Rodríguez et al., 2019; Fay et al., 2002; Seuma et al., 2022; Shuaib et al., 2020), which could explain the observed decrease in apoptosis. In *C. elegans*, treatment of the control strain CL802 with the Aβ_42_ variants and the WT peptide was not toxic to the nematodes. Although feeding Aβ-expressing *E. coli* has been shown to induce damage to the intestinal membrane (Hassan et al., 2015; Julien et al., 2018; Shea et al., 2019), systemic effects have not been reported.

Autophagy deregulation also contributes to Aβ toxicity in AD pathology (Nixon et al., 2005). Some studies have found that treating cells with Aβ_42_ activates autophagy by decreasing mTOR signaling and increasing LC3 levels, a human homologue of ATG8 (Chan et al., 2018; de la Cueva et al., 2022; Hu et al., 2009; Pomilio et al., 2020). Chronic exposure to Aβ_42_ impairs autophagy by disrupting lysosomal membranes (Jiang et al., 2018; Han et al., 2019; Pomilio et al., 2020), disrupting proteostasis (Caccamo et al., 2010), and inducing the accumulation of autophagosomes with eventual cell death (Jiang et al., 2018). Therefore, inhibition of autophagy could protect against Aβ_42_ toxicity in cell culture (Han et al., 2019; Jiang et al., 2018). However, other studies report conflicting results, showing an activation of mTOR and a decrease in autophagy in response to Aβ_42_ (Bhaskar et al., 2009; Caccamo et al., 2010). Under our conditions, we did not observe any change in autophagy in response to the WT peptide or its variants, suggesting that Aβ_42_ toxicity is dependent mainly on apoptosis in our cell model.

Similar dual effects have been observed in the worm, as in cell culture, with both activation and inhibition of autophagy providing protection against Aβ_42_ toxicity (Florez-McClure et al., 2007; Lin et al., 2022). We observed a decrease in GPF::LGG-1-positive puncta with M35C treatment in five- and ten-day AMH43 nematodes, and with A30W in 10-day worms. The number of LGG-1 puncta has been shown to increase with adulthood in pharynx, neurons, intestine, and muscle, reflecting decreased autophagy with aging (Chang et al., 2017). Although we quantified fewer puncta in 10-day worms, they appeared almost exclusively in the pharynx, as opposed to the 5-day nematodes, which presented signal mainly in the hypodermis. It is possible that the reduction in GPF::LGG-1 caused by A30W and M35C substitutions could reflect a protection against Aβ-induced autophagy dysfunction; however, more experiments are required to confirm this hypothesis.

In the context of Aβ_42_ constitutive expression, exposure to exogenous Aβ_42_ peptide increased toxicity in CL2006 nematodes. It’s known that aggregates of Aβ_42_ can act as seeds to accelerate the progression of amyloid pathology (Eisele, 2013; Liu et al., 2025; Sowade and Jahn, 2017; Walker et al., 2016). Similarly, nematodes expressing the amyloid protein α-synuclein in muscle show an increase in the number of aggregates after being fed with *E. coli* expressing a bacterial amyloid protein (Chen et al., 2016). Although we did not observe an increase in the number of amyloid deposits, the Aβ_42_ WT peptide may still contribute to the toxicity of the muscular Aβ_42_ expressed in CL2006 nematodes. On the contrary, the three variants showed a protective effect. We previously demonstrated that the A30W, K28A, and M35C Aβ_42_ variants can prevent and reverse the aggregation of the WT peptide (Estrada-Rodríguez et al., 2019). Consistently, we observed that treatment with the K28A and M35C peptides reduced the amount of fibrillar aggregates in CL2006 worms. Therefore, the protective effect of the K28A and M35C variants appears to be related to their anti-aggregation properties, as increased lifespan in CL2006 nematodes has been associated with a decrease in the number of Aβ_42_ deposits (Diomede et al., 2013; Yang J. et al., 2017; Zhang et al., 2016).

For the A30W variant, the extended lifespan in both CL2006 and CL802 strains, without any difference in the amount of fibrillar aggregates in the former, suggests that the peptide can affect aging independently of endogenous Aβ_42_. Some studies have found that protection against Aβ_42_ in*C. elegans* transgenic strains is primarily dependent on DAF-16 (Keowkase et al., 2010; Wang et al., 2016; Zhang et al., 2025). DAF-16 acts downstream of the insulin/insulin-like growth factor-1 signaling (IIS) pathway in the nematode. The reduction in IIS signaling leads to the translocation of DAF-16 to the nucleus, where it upregulates genes involved in the response to oxidative and heat stress, pathogens, and misfolded proteins, and downregulates genes associated with lipid and energy metabolism (Halaschek-Wiener et al., 2005; Lee, 2003). Therefore, this activation of DAF-16 protects the worm against stress and extends lifespan (Henderson and Johnson, 2001). A DAF-16 homologue in mammals, FOXO3A, has also been associated with longevity in humans, and a reduction in its serum levels has been found to correlate with AD risk, suggesting a possible interaction with Aβ and other factors linked to the disease (Pradhan et al., 2020; Du and Zheng, 2021; Liu et al., 2024). In *C. elegans*, the constitutive expression of Aβ can induce the overexpression of *daf-16* and its target genes (Godini et al., 2019; Gallrein et al., 2021; Lichty et al., 2025), creating a hormetic-like effect in which low doses of Aβ protect young nematodes against heat stress (Lichty et al., 2025). We hypothesize that this effect is enhanced in the A30W peptide, where exposure to this mutant induces DAF-16 activation, leading to protection against Aβ toxicity and lifespan extension. Additionally, it has been reported that the activity of DAF-16 in the intestine has the most weight in longevity when compared to other tissues (Libina et al., 2003; Uno et al., 2021), which relates to the nematodes being exposed to the peptides via feeding in our experiments. We did observe that the nuclear localization of DAF-16: GFP was more obvious in TJ356 worms after treatment with A30W; however, there was no significant difference in the number of nematodes with the cytosolic and intermediate phenotypes between our groups. Therefore, additional research is needed to characterize the mechanism by which the A30W variant induces lifespan extension in CL2006 and CL802 worms. Alternative techniques may be needed to detect changes in DAF-16 activity and other associated factors. Moreover, the peptide may exert its effects through other pathways not evaluated in this study, such as the AMP-activated protein kinase (AMPK) and the hypoxia inducible factor 1 (HIF-1), which are also related to lifespan regulation, and to a response against amyloid toxicity (Uno and Nishida, 2016; Xue et al., 2024; Lichty et al., 2025).

On the other hand, only the M35C and, unexpectedly, WT peptides were able to delay CL4176 paralysis. In these nematodes, toxicity is mainly related to Aβ oligomers, as paralysis occurs before any amyloid deposit can be detected (Drake et al., 2003). The methionine 35 residue is relevant in the formation of oligomers (Bitan et al., 2003; Durell et al., 2022); therefore, the M35C substitution could potentially reduce these toxic species. In the case of the WT peptide, it has been proposed that amyloid fibrillar aggregates can sequester soluble oligomers, potentially reducing their toxic effects (Gaspar et al., 2010; Yang T. et al., 2017), which could explain the observed protective effect.

The M35 amino acid is also relevant for Aβ-induced oxidative stress, as its oxidation can lead to the formation of a sulfuranyl radical cation (Butterfield and Sultana, 2011). Consistently, its substitution reduces ROS production in cell culture and *C. elegans* (Butterfield and Sultana, 2011; Estrada-Rodríguez et al., 2019; Yatin et al., 1999). We observed that M35C induced significantly less ROS than the A30W and WT peptides in the control CL802 nematodes, but not in CL4176 worms. M35C may exert a ROS-scavenging effect that is insufficient in the presence of constitutive Aβ42. The CL4176 strain shows a significant increase in protein oxidation after 32 h of muscular Aβ expression (Drake et al., 2003). In these worms, neither M35C nor WT altered Aβ-induced oxidative stress, whereas only K28A treatment significantly reduced ROS levels compared with the control. Our results show no correlation between oxidative stress and paralysis. Similarly, a rescue of paralysis induced by the *Ginkgo biloba* extract EGb 761 is related to the number of oligomers and not to the levels of H_2_O_2_ reactive species (Wu et al., 2006).

Overall, our findings showed that the A30W, K28A, and M35C substitutions reduce the toxicity of the Aβ_42_ peptide in a glioma cell line and in *C. elegans*. Additionally, treatment with the variants protected CL2006 worms by reducing Aβ_42_ fibrillar aggregation or by altering aging, whereas the M35C peptide protected CL4176 nematodes against toxic oligomers. The present work has limitations, including a small sample size in some of the experimental assays. Furthermore, the A30W peptide data indicate that rather than directly interacting with Aβ, which our methods cannot detect, our treatments may cause a systemic effect on the nematode. Despite these, this study contributes to understanding the influence of these residues on Aβ_42_ toxicity. Future research should seek to dissect the molecular mechanisms underlying the observed responses in control nematodes and Aβ-expressing strains. Additionally, the complete characterization of the peptides distribution in the worm from uptake should be sought. These approaches could help in the application of the A30W, K28A, and M35C peptides for future development of specific treatments for AD.

## Data Availability

The raw data supporting the conclusions of this article will be made available by the authors, without undue reservation.
